# Estimating the Force of Infection with *Helicobacter pylori* in Japan

**DOI:** 10.1155/2019/1451490

**Published:** 2019-01-30

**Authors:** Taishi Kayano, Ki-Deok Lee, Hiroshi Nishiura

**Affiliations:** ^1^Graduate School of Medicine, Hokkaido University, Kita 15 Jo Nishi 7 Chome, Kitaku, Sapporo 0608638, Japan; ^2^Department of Infectious Diseases, School of Medicine, Eulji University, Seoul 01830, Republic of Korea

## Abstract

**Background:**

Although the seroprevalence against *Helicobacter pylori* (*H. pylori*) in Japan has declined over the birth year, Japanese people have yet exhibited a relatively high risk of gastric cancer. The present study employed mathematical models to estimate the time- and age-dependent force of infection with *H. pylori* in Japan, predicting the future seroprevalence by time and age.

**Methods:**

We investigated the published seroprevalence data against *H. pylori* in Japan from 1980–2018. Solving the McKendrick partial differential equation model, the seroprevalence was modeled as a function of survey year and age. Maximum likelihood estimation was conducted to estimate parameters governing the time- and age-dependent force of infection.

**Results:**

Among all fitted models, the time-dependent and age-independent model with an exponentially decaying force of infection over years was most favored. Fitted models indicated that the force of infection started to decrease during and/or shortly after the World War II. Using the parameterized model, the predicted fraction seropositive at the age of 40 years in 2018 was 0.22, but it is expected to decrease to 0.13 in 2030 and 0.05 in 2050, respectively.

**Conclusion:**

The time dependence was consistent with the decline in the force of infection as a function of the birth year. The force of infection has continuously and greatly declined over time, implying the diminished transmission of *H. pylori* through the time course and small chance of persistence. These findings are critical to anticipate the future decline in gastric cancer incidence.

## 1. Introduction

*Helicobacter pylori* (*H. pylori*) is a bacterium known as the most important cause of gastric ulcer and cancer [1]. The bacterium is a helix-shaped gram-negative microaerophilic curved rod with four to six flagella at the same location, which is most commonly found in the stomach [2]. Although the exact mode of transmission has yet to be clarified, it is believed that the fecal-oral and/or oral-oral route are the most likely routes of transmission. A majority of people infected with *H. pylori* do not exhibit any clinical signs or symptoms. During the acute phase of infection, the symptoms associated with acute gastritis may occur. Moreover, chronic gastritis can lead to clinical symptoms that are associated with nonulcer dyspepsia. The long-lasting natural history of inflammation caused by chronic and atrophic gastritis is thought to be followed by carcinogenesis, and thus, the gastric cancer. The pathogenic factors of carcinogenesis such as VacA and CagA have been identified, and host genetic factors and several cytokine networks are proposed as the pathophysiological mechanism of cancer [3].

In the 21st century, the incidence of gastric cancer in Japan has continuously declined over time in all age groups. However, recent global estimates have indicated that Japanese people have yet exhibited a relatively high risk of gastric cancer [4], while the incidence of gastric cancer in developed regions have steadily declined. As an underlying explanation of the high incidence of gastric cancer, a high prevalence of *H. pylori* in the elderly in Japan has been considered as consistent with the natural history [1]. On the other hand, in many countries with decreased gastric cancer incidence over time, the seroprevalence of *H. pylori* has abruptly declined in young age cohorts [5].

A multi-institutional study across Japan, conducted by Ueda and his colleagues [6], has demonstrated a monotonic decline of *H. pylori* seroprevalence by birth cohorts. Similarly, analyzing the annual health check-up data, Hirayama et al. [7] have shown a continuous decline in *H. pylori* seroprevalence in Japan, e.g., a decline from 46 % among those born in 1940s to 18 % among those born in 1970s. Moreover, another published clinical epidemiologic study indicated that the seroprevalence in Japanese children was less than 2% [8]. Considering that the route of transmission with *H. pylori* is likely associated with direct contact and hygienic conditions during the childhood, decreased contact with environment in early ages (e.g., reduced chance to swim in the pond and reduced chance of parent-to-child transmission via bathroom) may have occurred, leading to the decreased seroprevalence of *H. pylori* even among adults.

While numerous studies have already reported the decreased seroprevalence of *H. pylori* and also decreased incidence of gastric cancer, the hazard rate or the time- and age-dependent risk of infection with *H. pylori* has yet to be explicitly reconstructed from the seroepidemiological data. Published series of cross-sectional seroepidemiological studies in Japan offer a unique opportunity to estimate the so-called force of infection, i.e., the rate at which susceptible individuals are infected. The present study aims to devise mathematical models to estimate the time- and age-dependent force of infection with *H. pylori* in Japan, elucidating the transmission dynamics in the past and predicting the future seroprevalence by time and age.

## 2. Method

### 2.1. Epidemiological Data

We investigated the seroprevalence data against *H. pylori* in Japan from 1980–2018. Although the present study does not strictly adhere to the statement and officially acknowledged methodological details of the systematic review, the following literature review was systematically conducted, searching the MEDLINE and Web of Science databases, using the following search terms:

  “Seroprevalence OR Seroepidemic OR Seropositive OR Serological OR Serosurvey OR
  IgG,
  AND Helicobacter OR *H. pylori*
  AND Japan.”

All titles identified by the search strategy were independently screened by two authors (TK and HN). Abstracts of potentially relevant titles were then reviewed for eligibility, and articles were selected for closer evaluation, if a description of the seroepidemiological study of *H. pylori* among the Japanese was available. Clinical and epidemiological studies that rested on laboratory methods other than serology and that offered nontractable seroepidemiological data over time or age were excluded. Before the present study, there was a narrative review article by Inoue [1], and we added to our literature those cited in the review but were missed by the above-mentioned systematic search.

### 2.2. Time and Age Elements

From each included paper, we extracted the information over the number of positive/negative samples by survey year and age. When the original data yielded the age information as only discrete age groups, we used the midpoint of age for modeling purpose. The birth year was calculated as survey year minus age. As the total number of samples and the count of positive samples are available, we computed the 95% confidence intervals (CI) of observed seroprevalence, using a binomial distribution.

### 2.3. Mathematical Model

The observed seropositive fraction represents the time- and age-specific history of the past exposure. We employ a mathematical model to capture the time- and age-dependent transmission dynamics of *H. pylori* from the seroprevalence data, and in particular, the present study jointly explores the time at which the rate of infection, *t*_0_ started to decrease. Let *s*(*a*, *t*) be the fraction of susceptible individuals at age *a* and year *t*. Assuming that everyone is born susceptible to *H. pylori* and discarding maternal antibodies, the boundary condition would be *s*(0, *t*)=1 for any *t*. Let *λ*(*a*, *t*) be the force of infection, i.e., the rate at which susceptible individuals experience infection, which depends on age *a* and year *t*, the susceptible individuals are depleted by

(1) $$\begin{array}{c} \left(\frac{\partial }{\partial a}+\frac{\partial }{\partial t}\right)s\left(a,\;\;t\right)=-\lambda \left(a,t\right)s\left(a,\;\;t\right). \end{array}$$

We assume that the force of infection is separable to age- and time-components, i.e.,

(2) $$\begin{array}{c} \lambda \left(a,t\right)=f\left(a\right)g\left(t\right). \end{array}$$

Integrating both sides of equation (1) along the characteristic line, we obtain

(3) $$\begin{array}{c} s\left(a,\;\;t\right)=s\left(0,\;\;t-a\right)\exp\left(-\int_{t-a}^{t}\lambda \left(y-t+a,y\right)\;dy\right),\\ =\exp\left(-\int_{t-a}^{t}f\left(y-t+a\right)g\left(y\right)\;dy\right), \end{array}$$

for *t* > *a*. Using the susceptible fraction, *s*(*a*, *t*), the seroprevalence at age *a* and in year *t* is obtained from 1 − *s*(*a*, *t*). To quantify the force of infection, we impose three different parametric assumptions. First, we assume that *g*(*t*) was initially a constant, *λ*_0_, but from the year *t*_0_ has been exponentially decreasing with year *t*, i.e., *g*(*t*)=*λ*_0_ exp(−*δ*(*t* − *t*_0_)), and also that the force of infection is age-independent. We have the seroprevalence, *p*(*a*, *t*), parameterized as

(4) $$\begin{array}{c} p\left(a,\;\;t\right)=\left\{\begin{array}{cc} 1-\exp\left(-\int_{t-a}^{t}\lambda_{0}\exp\left(-\delta \left(s-t_{0}\right)\right)\;ds\right), & \text{for}\;\;t-a\geq t_{0},\\ 1-\exp\left(-\lambda_{0}\left(t_{0}-t+a\right)-\int_{t_{0}}^{t}\lambda_{0}\exp\left(-\delta \left(s-t_{0}\right)\right)\;ds\right), & \text{for}\;\;t-a<t_{0}. \end{array}\right. \end{array}$$

Hereafter, we identify this model as model 1.

Alternatively, following the year *t*_0_, we assume that *g*(*t*) has experienced a time-dependent decay that follows the Gompertz law with year *t*, i.e., *g*(*t*)=*λ*_0_ exp(−*β*(exp(*γ*(*t* − *t*_0_)) − 1)), and also that the force of infection is age-independent. We identify it as model 2 and we have

(5) $$\begin{array}{c} p\left(a,t\right)=\left\{\begin{array}{cc} 1-\exp\left(-\int_{t-a}^{t}\lambda_{0}\exp\left(-\beta \left(\exp\left(\gamma \left(s-t_{0}\right)\right)-1\right)\right)\;ds\right), & \text{for}\;\;t-a\geq t_{0},\\ 1-\exp\left(-\lambda_{0}\left(t_{0}-t+a\right)-\int_{t_{0}}^{t}\lambda_{0}\exp\left(-\beta \left(\exp\left(\gamma \left(s-t_{0}\right)\right)-1\right)\right)\;ds\right), & \text{for}\;\;t-a<t_{0}. \end{array}\right. \end{array}$$

As model 3, we assume that *g*(*t*) has experienced a time-dependent exponential decay following *t*_0_, *g*(*t*)=*λ*_0_exp(−*δ*(*t* − *t*_0_)), and throughout the course of time, we also assume an age-dependent exponential decay, i.e., *f*(*a*)=exp(−*ρa*). The seroprevalence of model 3 is described as

(6) $$\begin{array}{c} p\left(a,\;\;t\right)=\left\{\begin{array}{cc} 1-\exp\left(-\int_{t-a}^{t}\lambda_{0}\exp\left(-\delta \left(s-t_{0}\right)\right)\exp\left(-\rho \left(s-t+a\right)\right)\;ds\right), & \text{for}\;\;t-a\geq t_{0},\\ 1-\exp\left(-\int_{t-a}^{t_{0}}\lambda_{0}\exp\left(-\rho \left(s-t+a\right)\right)\;ds\;-\int_{t_{0}}^{t}\lambda_{0}\exp\left(-\delta \left(s-t_{0}\right)\right)\exp\left(-\rho \left(s-t+a\right)\right)\;ds\right), & \text{for}\;\;t-a<t_{0}. \end{array}\right. \end{array}$$

In the above-mentioned models, *λ*_0_, *δ*, *t*_0_, *β*, and *ρ* are dealt with as parameters to be estimated. To quantify the force of infection by estimating those parameters, we employed a likelihood-based approach. Given that there were *m*_*a*,*t*_ positive individuals among the total of serum samples drawn from *n*_*a*,*t*_ individuals in age *a* and year *t*, the likelihood function to estimate parameter *θ* was modeled as

(7) $$\begin{array}{c} L\left(\theta :\;\;n,m\right)=\underset{a}{\prod }\underset{t}{\prod }\left(\begin{array}{c} n_{a,t}\\ m_{a,t} \end{array}\right)p{\left(a,t\right)}^{m_{a,t}}{\left(1-p\left(a,t\right)\right)}^{n_{a,t}-m_{a,t}}. \end{array}$$

The 95% CI of parameters were computed using the profile likelihood. Once all parameters are estimated, we calculated the predicted seroprevalence as a function of time and age, especially, in years 2018, 2030, and 2050 for the exposition of the advantage of our approach to estimate the force of infection.

## 3. Results

**Figure 1:**
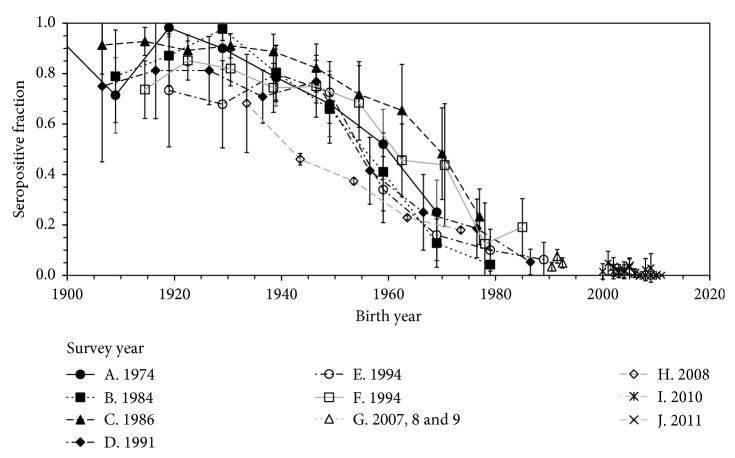
Seroprevalence of anti-*Helicobacter pylori* antibody in Japan by birth year. Antibody positive fraction is reviewed as a function of birth year. Same marks represent the dataset arising from an identical publication in the same survey year. Whiskers extend to lower and upper 95% confidence intervals.

In total, 10 seroprevalence studies were identified and used in the following analyses (Figure 1) [1, 5, 7–17]. Excluded seroepidemiological and clinical studies from the following analyses are a cohort study with variable timing of seroprevalence surveys that cannot be traced back from the literature [5], a clinical study that used urinary samples for testing *H. pylori* [17], and a study that missed the information of age grouping in summarizing seroepidemiological datasets [15]. All included publications measured the *H. pylori*-IgG antibody at the population level in a cross-sectional manner by age groups. The identified oldest survey took place in 1974, while the latest study was conducted in 2011. A small number of studies focused on particular age groups, especially on children, recruiting those aged from 0–11 years [9] and those at high school [14]. Figure 1 reveals that the seroprevalence drastically declined with birth year, and thus, most likely with time. The figure also indicated that the sample size was not large enough to distinguish the seroprevalence in one study from others in nearby birth cohorts, involving many overlapping confidence intervals of seroprevalence. Moreover, for each birth year, the expected value of the seroprevalence did not evidently increase according to the order of survey year, implying a limited age effect.

**Figure 2:**
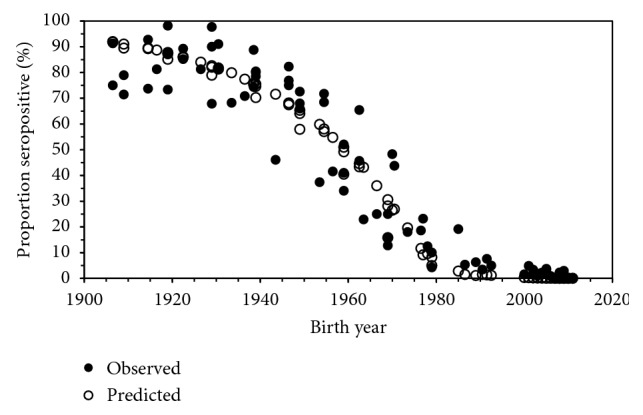
Comparison between observed and predicted seroprevalence against *Helicobacter pylori* in Japan by birth year. Observed data (filled marks) in various surveys are plotted by birth year and compared against model prediction (unfilled marks) that assumes time dependence in the force of infection with an exponential decay. Predictions were made as a function of survey year and age.

Fitting three different models to the identified data, we compared the goodness of fit as informed by the Akaike information criterion (AIC) (Table 1). Among all three models, including those employing the time-dependent and time- and age-dependent forces of infection, the model 1 with an exponential decay of the force of infection with time and without age dependence yielded the minimum AIC and was considered as the best fit model.  Figure 2 compares the observed and predicted seroprevalence data by birth year, confirming that the observed patterns were overall well captured by the model 1. Due to the absence of age dependence, the predicted value did not vary over the vertical axis in Figure 2.

**Figure 3:**
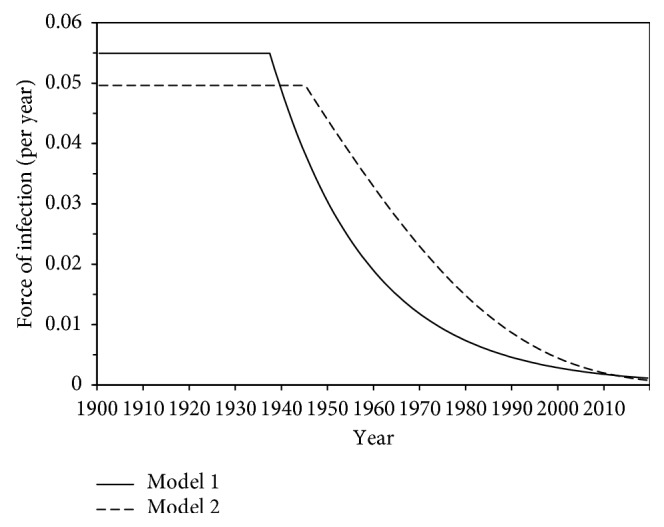
Estimated force of infection of *Helicobacter pylori* as a function of calendar time in Japan. Model 1 (bold straight line) is the estimate of time-dependent force of infection with an exponential decay. Model 2 (dashed line) is the estimate of time-dependent force of infection with Gompertz-type decay.

Figure 3 shows the estimated force of infection with *H. pylori* as a function of calendar time in Japan, using models 1 and 2. Qualitatively, the predicted values of models 1 and 2 were close to each other. The estimated year in which the force of infection was considered to have started to decrease was estimated at 1937 in model 1, while that of model 2 was 1945, the year corresponding to the end of the World War II. Estimated parameters of the best fitted model 1, *λ*_0_, *δ*, and *t*_0_ were 0.056 (95% CI: 0.048, 0.065) per year, 0.047 (95% CI: 0.045, 0.050) per year, and 1937 (95% CI: 1933, 1940), respectively.

**Figure 4:**
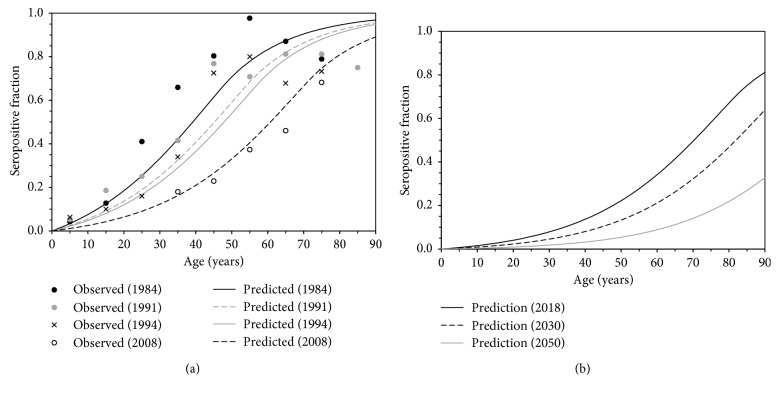
Prediction of the seroprevalence against *Helicobacter pylori* in the past and the future in Japan. (a) Comparison between observed and predicted seroprevalence by age and survey year. Marks represent observed data, while lines are the expected values derived from the time-dependent force of infection with an exponential decay. (b) Prediction of the future seroprevalence against *Helicobacter pylori* in Japan. Gradual right shift in the seroprevalence is captured by our time-dependent force of infection with an exponential decay.

Figure 4 shows the seroprevalence against *H. pylori* as a function of age in the past and the future in Japan. In Figure 4(a), the seroprevalence has exhibited a sigmoidal shape to increase as a function of age. While a part of the past observed data look not well aligned with the predicted values (e.g., those in 1984), the observed points with small serological samples suffered from broad uncertainty (i.e. wide confidence intervals), and the predicted values in general well captured the observed patterns of the seroprevalence data by time and age. When the future seroprevalence was predicted (Figure 4(b)), the seroprevalence revealed a clear pattern of right shift over age by the year of prediction (i.e. the elevation of age at infection over future years is anticipated). For instance, the predicted fraction seropositive at the age of 40 years in 2018 would be 0.22, but it is expected to decrease to 0.13 in 2030 and 0.05 in 2050, respectively.

## 4. Discussion

The present study explored the long-term dynamics of *H. pylori* infection in Japan, estimating the force of infection from a total of 10 different seroepidemiological survey datasets. Fitting time-dependent and time- and age-dependent models, the time-dependent force of infection with an exponential decline was selected as the best fit model. Fitted models indicated that the force of infection started to decrease during and/or shortly after the World War II. Subsequently, the force of infection was considered to have steadily declined over time. Using the parameterized model, the age of seropositive individuals was predicted to be greatly shifted to older groups in the future, which would be more evident than in the past. To our knowledge, the present study is the first to elucidate the time-dependent dynamics of *H. pylori* in Japan, offering predictions of seropositivity in 2030 and 2050.

The time dependence was consistent with the decline in the force of infection as a function of birth year (Figure 1) and without many variations over vertical axis, reflecting limited variations over age. Such decline with birth year has also been seen in other settings, e.g., China [18]. Using the mathematical model and the force of infection, the present study achieved to translate the decreasing pattern into the time-dependent decline in the hazard rate of infection and also permitted the future prediction of seroprevalence. The present study endorses the long-lasting notion of expert on this subject (e.g., a hypothesis by Blaser [19]): the seroprevalence continuously and greatly declines over time, implying the diminished transmission over time and small chance of persistence. These findings are critical to anticipate the future decline in gastric cancer, which have already been studied using mathematical models [20, 21].

While the time- and age-dependent model was not selected as the best model, it should be noted that the present study did not exclude the possibility that there is strong age dependence in the force of infection, especially among young children. In fact, the age effect was strongly indicated in the literature [22–26] and, as we noted from Figure 1, the uncertainty bound of seroprevalence in each birth year was very broad and the sampling error was not avoidable. Both empirical and theoretical studies clearly demonstrate that the infection mostly occurs in children [22, 23], and this notion is consistent with our age-dependent term of model 3, i.e., as a function of age, and the force of infection was considered to have decreased exponentially with the rate of 0.57 per year, indicating that the average age at infection was about 2 years. The infections would therefore perhaps be mostly seen among children, and additional studies with more precise observation with greater sample size would be essential to better identify the age dependence.

Apart from the predicted future course of the seroprevalence, it is natural to wonder how the epidemiology of gastric cancer will behave in the future. Not only the seroprevalence but also the future demographic dynamics, e.g., aging and decreasing population, would be a highly influential factor in regulating the incidence of cancer in the future. Estimating the induction period, i.e., the time from exposure to *H. pylori* to the cancer development, the future incidence of gastric cancer will become predictable by employing a mathematical modeling approach. In line with this intent, another interesting direction is to explore the epidemiological impact of eradication therapy of *H. pylori* on the gastric cancer incidence in an explicit manner.

Four limitations must be noted. First, the present study rested on the review of collected seroprevalence studies in different geographic locations. Depending on the geographic region in Japan, environmental conditions that can lead to infection can be different (e.g., urban vs. rural), but we collectively analyzed all the datasets as serial cross-sectional data in a single population. Second, even though we performed the systematic search of literature and minimized the sampling error, the sample size was small for each age group in each individual study, and the sampling error was substantial in quantifying the age effect. Third, fixed cutoffs used by a serological assay might have resulted in underascertainment of seropositive individuals, and it can sacrifice specificity, while sensitivity is ensured to be high [27]. Fourth, the force of infection is sometimes modeled as a function of the prevalence of infectious individuals, but the present study did not fully disentangle the underlying transmission dynamics by decomposing the force of infection into multiple mathematical functions.

Besides, without proper use of mathematical models, one cannot clarify the hazard rate of infection and predict the future seroprevalence [28–33]. In many countries, the decline in seroprevalence has been observed over birth cohort [1, 4, 5, 22, 34], and the present study uniquely and successfully identified the strong signature of the time dependence in the force of infection in Japan. To further predict the future size of gastric cancer, we trust that the present study substantially contributes to building the foundation for such sophisticated exercise.

## Figures and Tables

**Table 1:** Model comparison of the time- and age-dependent force of infection to capture the transmission dynamics of *Helicobacter pylori* in Japan.

Model identity	Functional assumption	Number of parameters	AIC
Model 1	Time-dependent FOI with an exponential decay	3	937.2
Model 2	Time-dependent FOI with a Gompertz-type decay	4	3856.3
Model 3	Time- and age-dependent FOI with an exponential time-decay and exponential age-decay	4	2750.5

AIC: Akaike information criterion; FOI: force of infection.

## Data Availability

The data used to support the findings of this study are available from the corresponding author upon request.

## References

[1] Inoue M. (2016). Changing epidemiology of *Helicobacter pylori* in Japan. *Gastric Cancer*.

[2] Marshall B., Warren J. R. (1984). Unidentified curved bacilli in the stomach of patients with gastritis and peptic ulceration. *The Lancet*.

[3] Chiba T., Marusawa H., Seno H., Watanabe N. (2008). Mechanism for gastric cancer development by *Helicobacter pyloriinfection*. *Journal of Gastroenterology and Hepatology*.

[4] Hooi J. K. Y., Lai W. Y., Ng W. K. (2017). Global prevalence of *Helicobacter pylori* infection: systematic review and meta-analysis. *Gastroenterology*.

[5] Watanabe M., Ito H., Hosono S. (2015). Declining trends in prevalence of *Helicobacter pyloriinfection* by birth-year in a Japanese population. *Cancer Science*.

[6] International Agency for Research on Cancer (IARC) (2014). IARC *Helicobacter pylori* Working Group. *Helicobacter pylori* Eradication as a strategy for preventing gastric cancer. *IARC Working Group Reports*.

[7] Ueda J., Gosho M., Inui Y. (2014). Prevalence of *Helicobacter pyloriInfection* by birth year and geographic area in Japan. *Helicobacter*.

[8] Hirayama Y., Kawai T., Otaki J., Kawakami K., Harada Y. (2014). Prevalence of *Helicobacter pyloriinfection* with healthy subjects in Japan. *Journal of Gastroenterology and Hepatology*.

[9] Okuda M., Osaki T., Lin Y. (2014). Low prevalence and incidence of *Helicobacter pyloriInfection* in children: a population-based study in Japan. *Helicobacter*.

[10] Fujisawa T., Kumagai T., Akamatsu T., Kiyosawa K., Matsunaga Y. (1999). Changes in seroepidemiological pattern of *Helicobacter pylori* and hepatitis A virus over the last 20 years in Japan. *The American Journal of Gastroenterology*.

[11] Malaty H. M., Tanaka E., Kumagai T. (2003). Seroepidemiology of *Helicobacter pylori* and hepatitis A virus and the mode of transmission of infection: a 9-year cohort study in rural Japan. *Clinical Infectious Diseases*.

[12] Kumagai T., Malaty H. M., Graham D. Y. (1998). Acquisition versus loss of *Helicobacter pyloriInfection* in Japan: results from an 8-year birth cohort study. *Journal of Infectious Diseases*.

[13] Asaka M., Kimura T., Kudo M. (1992). Relationship of *Helicobacter pylori* to serum pepsinogens in an asymptomatic Japanese population. *Gastroenterology*.

[14] Akamatsu T., Ichikawa S., Okudaira S. (2011). Introduction of an examination and treatment for *Helicobacter pylori* infection in high school health screening. *Journal of Gastroenterology*.

[15] Ueda J., Gosho M., Inui Y. (2014). Prevalence of *Helicobacter pyloriInfection* by birth year and geographic area in Japan. *Helicobacter*.

[16] Shiota S., Murakami K., Fujioka T., Yamaoka Y. (2014). Population-based strategies for *Helicobacter pylori*-associated disease management: a Japanese perspective. *Expert Review of Gastroenterology and Hepatology*.

[17] Tamura T., Morita E., Kondo T. (2012). Prevalence of *Helicobacter pylori* infection measured with urinary antibody in an urban area of Japan, 2008-2010. *Nagoya Journal of Medical Science*.

[18] Yu X., Yang X., Yang T., Dong Q., Wang L., Feng L. (2017). Decreasing prevalence of *Helicobacter pylori* according to birth cohorts in urban China. *The Turkish Journal of Gastroenterology*.

[19] Blaser M. J. (1999). Hypothesis: the changing relationships of *Helicobacter pyloriand* humans: implications for health and disease. *Journal of Infectious Diseases*.

[20] Gershengorn H. B., Blower S. M., Castillo-Chavez C. (2001). Modeling cancer as an infectious disease: the epidemiology of *Helicobacter pylori*. *Mathematical Approaches for Emerging and Reemerging Infectious Diseases*.

[21] Rupnow M., Shachter R. D., Owens D. K., Parsonnet J. (2000). A dynamic transmission model for predicting trends in *Helicobacter pylori* and associated diseases in the United States. *Emerging Infectious Diseases*.

[22] Malaty H. M., El-Kasabany A., Graham D. Y. (2002). Age at acquisition of *Helicobacter pylori* infection: a follow-up study from infancy to adulthood. *The Lancet*.

[23] Alarid-Escudero F., Enns E. A., MacLehose R. F., Parsonnet J., Torres J., Kuntz K. M. (2018). Force of infection of *Helicobacter pylori* in Mexico: evidence from a national survey using a hierarchical Bayesian model. *Epidemiology and Infection*.

[24] Veldhuyzen van Zanten S. J. O., Pollak P. T., Best L. M., Bezanson G. S., Marrie T. (1994). Increasing prevalence of *Helicobacter pylori* infection with age: continuous risk of infection in adults rather than cohort effect. *Journal of Infectious Diseases*.

[25] Rowland M., Daly L., Vaughan M., Higgins A., Bourke B., Drumm B. (2006). Age-specific incidence of *Helicobacter pylori*. *Gastroenterology*.

[26] Zabala Torrres B., Lucero Y., Lagomarcino A. J. (2017). Review: prevalence and dynamics of *Helicobacter pylori* infection during childhood. *Helicobacter*.

[27] Kafatos G., Andrews N. J., McConway K. J., Maple P. A. C., Brown K., Farrington C. P. (2015). Is it appropriate to use fixed assay cut-offs for estimating seroprevalence?. *Epidemiology and Infection*.

[28] Hamaguchi Y., Yamaguchi T., Nishiura H. (2019). Estimating the annual risk of tuberculosis infection in Japan from interferon-gamma release assay data. *Journal of Theoretical Biology*.

[29] Yuan B., Nishiura H. (2018). Estimating the actual importation risk of dengue virus infection among Japanese travelers. *PLoS One*.

[30] Nabae K., Satoh H., Nishiura H. (2014). Estimating the risk of parvovirus B19 infection in blood donors and pregnant women in Japan. *PLoS One*.

[31] Yoshii K., Kojima R., Nishiura H. (2017). Unrecognized subclinical infection with tickborne encephalitis virus, Japan. *Emerging Infectious Diseases*.

[32] Kinoshita R., Nishiura H. (2017). Assessing age-dependent susceptibility to measles in Japan. *Vaccine*.

[33] Kinoshita R., Nishiura H. (2016). Assessing herd immunity against rubella in Japan: a retrospective seroepidemiological analysis of age-dependent transmission dynamics. *BMJ Open*.

[34] Roberts S. E., Morrison-Rees S., Samuel D. G., Thorne K., Akbari A., Williams J. G. (2015). Review article: the prevalence of *Helicobacter pyloriand* the incidence of gastric cancer across Europe. *Alimentary Pharmacology and Therapeutics*.

